# Miscellaneous

**Published:** 1892-09

**Authors:** 


### MISCELLANEOUS.

### WASHINGTON LETTER.

What has been done at the National Capital during the month in the interests of the doctor, surgeon, dentist, druggist, etc.

After my letter of last month closed, the homeopathic physicians transacted but little business before they adjourned to meet in Chicago next year. They adopted resolutions discountenancing the patent medicine man and providing for his expulsion from their society, endorsed the Paddock pure food bill and recommended its passage by the House, and denounced the chloride of gold cure as a humbug; and then went over to Baltimore to attend a reception by the Southern Homeopathic College.

The first week of this month was an insufferably hot one here and ran our death rate up from 143 last year to 152, or 15 more than in the week preceding. Of these deaths, 84 were children under 5 years of age, and 73 were under twelve months old. 44 deaths resulted from diarrheal causes, and many more were chargeable indirectly to the heat. Not one death resulted from malarial causes, which speaks well for our city in summer. We have a ship anchored down the Potomac, whereon all small pox patients are put; but I regret to say that thousands of excursionists pass this ship daily, and run the gauntlet of that dread malady. It should be stationed a few miles lower down beyond the path of the excursion boats. The following week, and each one since, shows an encouraging decrease in the death rate; but it will rise again when the real hot weather returns.

The Agricultural Department reports that the enforcement of the meat inspection law of March 3d, 1891, has added at least one per cent. a pound to the value of hogs marketed since the withdrawal of the foreign prohibition. The governments of the world are looking with great favor upon this stringent inspection of ours. The U. S. manufacturing Chemists' Association has protested against the bill to prevent the adulteration and misbranding of foods and drugs; and the N. J. Pharmaceutical Association has petitioned Congress to adopt the metric system of weights any measures in the customs service. With the first of July the city adopted a system of carrying off garbage in patent air-tight steel carts, and treating it with exhaust steam to pass the offensive gases into the sewers. The plan works well, and we are delighted.

The Capitol building has been examined by experts who report its sanitary condition as something awful; and the plumbing in the Supreme Court chamber as a "sanitary curiosity:" The plumbing is said to be from 40 to 90 years old, and even the Senate restaurant needs entire reconstruction. It will take about $90,000 to renew this plumbing entire, and Senator Vest has urged the spending of that sum, to be incorporated in one of the appropriation bills. The matter meets the approval of everybody, and no doubt will go through without opposition. It is expected the work will be done during the summer so that the Supreme Court which meets in October will have a clean and healthy home to work in, as well as both Houses of Congress when they reconvene the first of December.

### LEAD AND PRESERVED FOODS.

The presence of lead in the vessels used for preserved foods, and the danger thereby caused to the health of the consumer, is a matter demanding more attention than it has hitherto received. Lead is a poisonous metal of a particularly dangerous kind. It easily enters into combination with a variety of substances, and it does so, apparently, under a variety of conditions, forming compounds which are readily absorbed; while the well known "cumulative" property of the compound of lead, when steadily introduced into the system, even in exceedingly minute quantities, makes its introduction an especially insidious form of poisoning. According to some recently published results, it appears that in regard to the solder used for closing cans, the composition may vary at different parts of the same vessel, that used for finally closing the can, after sterilization, being usually richest in lead, on account of its greater fusibility and consequent ease of working. A can of American fruit was found to be "tinned" with an alloy containing 0.55 per cent. of lead, while its solder under the lid contained 50.84 per cent. of this metal. Another can containing corned beef was found to contain only traces of lead in the "tinning," while the solder under the lid was of the same composition as that above referred to, containing 50.06 per cent. of lead.

### SOMETHING TO PICK.

How many bones in the human face ?
Fourteen, when they're all in place.

How many bones in the human head ?
Eight. my child, as I've often said.

How many bones in the human ear ?
Three in each, and they help to hear.

How many bones in the human spine ?
Twenty-six, like a climbing vine.

How many bones in the human chest ?
Twenty-four ribs, and two of the rest.

How many bones in the shoulder bind ?
Two in each—one before and behind.

How many bones in the human arm ?
In each one, two in each forearm.

How many bones in the human wrist ?
Eight in each, if none are missed.

How many bones in the palm of the hand ?
Five in each, with many a band.

How many bones in the fingers ten ?
Twenty-eight, and by joints they bend.

How many bones in the human hip ?
One in each, like a dish they dip.

How many bones in the human thigh ?
One in each, and deep they lie.

How many bones in the human knees ?
One in each, the knee-pan, please.

How many bones in the ankle strong ?
Seven in each, but none are long.

How many bones in the ball of the foot ?
Five in each, as the palms were put.

How many bones in the toes half a score ?
Twenty-eight, and there are no more.

And now altogether, these many bones fix,
And they count in the body two hundred and six.

And then we have the human mouth,
Of upper and under thirty-two teeth.

And now and then have a bone, I should think
That forms on a joint, or to fill up a chink.

A sesamoid bone, or a wormain, we call,
And now we may rest, for we've told them all.—*Medical Recorder*.

An Affectionate Mother.—So many pathetic stories are told of the misery caused by hunters, one can scarcely bear the idea of shooting merely for "sport." When the term means merely wanton cruelty, then it is time to seek more peaceful if less exciting occupations. A story is told of a polar bear, which, with two large cubs, was sighted by the crew of an exploring frigate. When the animals were within reach of the vessel, the sailors threw them great lumps of sea-horse flesh, and these the old bear divided among her cubs, reserving only a small portion for herself. Then, when the three animals were happy feeding, the sailors fired. They wounded the dam and killed the cubs.

It would have drawn tears of pity from any but the unfeeling to have marked the affectionate concern expressed by this poor beast in the last moments of her expiring young. Though she was dreadfully wounded, she tore another lump of flesh to pieces, and laid it before them.

When she found that they would not eat, she laid her paws first on one and then the other, and tried to raise them up ; all this time it was pitiful to hear her moan. When she was convinced that they would not stir, she walked away, looking back and still moaning ; and when that did not entice them to rise, she returned and began to lick their wounds.

She went off a second time as before, and having crawled a few paces looked again behind her. The cubs did not rise, and she returned, and with signs of inexpressible fondness went round pawing them and moaning. Finding at last that they were cold and lifeless, she raised her head toward the ship and uttered a growl of despair which the murderers returned with a volley of balls. Then she fell between her cubs and died, licking their wounds.

